# Retraction Note: Systematic analyses reveal long non-coding RNA (PTAF)-mediated promotion of EMT and invasion-metastasis in serous ovarian cancer

**DOI:** 10.1186/s12943-023-01779-x

**Published:** 2023-04-28

**Authors:** Haihai Liang, Xiaoguang Zhao, Chengyu Wang, Jian Sun, Yingzhun Chen, Guoyuan Wang, Lei Fang, Rui Yang, Mengxue Yu, Yunyan Gu, Hongli Shan

**Affiliations:** 1 Department of Pharmacology (State-Province Key Laboratories of Biomedicine-Pharmaceutics of China, Key Laboratory of Cardiovascular Research, Ministry of Education), College of Pharmacy, Harbin Medical University, Translational Medicine Research and Cooperation Center of Northern China, Heilongjiang Academy of Medical Sciences, Harbin, 150081 China; 2 Department of Systems Biology, College of Bioinformatics Science and Technology, Harbin Medical University, Harbin, 150001 China; 3 Department of Pathology of The Second Affiliated Hospital, Harbin Medical University, Harbin, 150081 China; 4 Department of Pathology of The First Affiliated Hospital, Harbin Medical University, Harbin, 150081 China; 5 Department of Obstetrics and Gynecology, The Second Affiliated Hospital of Harbin Medical University, Harbin, 150081 China; 6 Department of Systems Biology, College of Bioinformatics Science and Technology, Harbin Medical University, Harbin, 150086 China; 7 Training Center for Students Innovation and Entrepreneurship Education, Harbin Medical University, Harbin, 150086 China

**Retraction Note: Mol Cancer 17, 96 (2018)**

**https://doi.org/10.1186/s12943-018-0844-7**

The Editor-in-Chief has retracted this article. Following the correction of this article, concerns were raised regarding the corrected Fig. 6: the 0 h PTAF + miR-25 panel in the corrected Fig. 6a appears to partially overlap with the 0 h shPTAF + AMO-25 panel of the corrected Fig. 6d. Additionally, concerns were raised regarding Supplementary Fig. 3D: the panels for PTAF and PTAF + miR-Ctrl appear to partially overlap. The Editor-in-Chief therefore no longer have confidence in the results and conclusions of this article.

Author Yunyan Gu has stated on behalf of all authors that they agree with this retraction.
